# Strong Signs for a Weak Wall in Tricuspid Aortic Valve Associated Aneurysms and a Role for Osteopontin in Bicuspid Aortic Valve Associated Aneurysms

**DOI:** 10.3390/ijms20194782

**Published:** 2019-09-26

**Authors:** Christian Stern, Bernhard Scharinger, Adrian Tuerkcan, Clemens Nebert, Teresa Mimler, Ulrike Baranyi, Christian Doppler, Thomas Aschacher, Martin Andreas, Marie-Elisabeth Stelzmueller, Marek Ehrlich, Alexandra Graf, Guenther Laufer, David Bernhard, Barbara Messner

**Affiliations:** 1 Cardiac Surgery Research Laboratory, Department of Cardiac Surgery, Medical University of Vienna, 1090 Vienna, Austria; Christian.stern@medizin.uni-halle.de (C.S.); be.scharinger@salk.at (B.S.); atuerkcan@gmail.com (A.T.); clemens.nebert@meduniwien.ac.at (C.N.); teresa.mimler@meduniwien.ac.at (T.M.); ulrike.baranyi@meduniwien.ac.at (U.B.); thomas.aschacher@meduniwien.ac.at (T.A.); 2 Julius-Bernstein-Institute for Physiology, Medical Faculty of the Martin-Luther- University, 06112 Halle-Wittenberg, Germany; 3 Department of Radiology, Paracelsus Medical University Salzburg, 5020 Salzburg, Austria; 4 Cardiac Surgery Research Laboratory, University Clinic for Cardiac Surgery, Medical University of Innsbruck, 6020 Innsbruck, Austria; christian.doppler@jku.at (C.D.); david.bernhard@jku.at (D.B.); 5 Division for Pathophysiology, Institute of Physiology and Pathophysiology, Johannes Kepler University Linz, 4020 Linz, Austria; 6 Department of Surgery, Cardiac Surgery, Medical University of Vienna, 1090 Vienna, Austria; martin.andreas@meduniwien.ac.at (M.A.); marie-elisabeth.stelzmueller@meduniwien.ac.at (M.-E.S.); marek.ehrlich@meduniwien.ac.at (M.E.); guenther.laufer@meduniwien.ac.at (G.L.); 7 Center for Medical Statistics, Informatics and Intelligent Systems, Medical University of Vienna, 1090 Vienna, Austria; alexandra.graf@meduniwien.ac.at

**Keywords:** osteopontin, alpha smooth muscle actin, focal elastic fiber loss, atherosclerosis, bicuspid, tricuspid

## Abstract

Central processes in the pathogenesis of TAV- (tricuspid aortic valve) and BAV- (bicuspid aortic valve) associated ascending thoracic aortic aneurysm (ATAA) development are still unknown. To gain new insights, we have collected aortic tissue and isolated smooth muscle cells of aneurysmal tissue and subjected them to in situ and in vitro analyses. We analyzed aortic tissue from 78 patients (31 controls, 28 TAV-ATAAs, and 19 BAV-ATAAs) and established 30 primary smooth muscle cell cultures. Analyses included histochemistry, immuno-, auto-fluorescence-based image analyses, and cellular analyses including smooth muscle cell contraction studies. With regard to TAV associated aneurysms, we observed a strong impairment of the vascular wall, which appears on different levels—structure and dimension of the layers (reduced media thickness, increased intima thickness, atherosclerotic changes, degeneration of aortic media, decrease of collagen, and increase of elastic fiber free area) as well as on the cellular level (accumulation of fibroblasts/myofibroblasts, and increase in the number of smooth muscle cells with a reduced alpha smooth muscle actin (α-SM actin) content per cell). The pathological changes in the aortic wall of BAV patients were much less pronounced—apart from an increased expression of osteopontin (OPN) in the vascular wall which stem from smooth muscle cells, we observed a trend towards increased calcification of the aortic wall (increase significantly associated with age). These observations provide strong evidence for different pathological processes and different disease mechanisms to occur in BAV- and TAV-associated aneurysms.

## 1. Introduction 

With 60% of all dilatations of the thoracic aorta, ascending aortic aneurysms are the most common form of aneurysm affecting this region [1]. The majority of ascending thoracic aortic aneurysm (ATAA) development is of unknown origin and is therefore classified as idiopathic or sporadic forms [1,2,3]. A known risk factor for the development of ATAAs is the presence of the most common congenital cardiac malformation, namely the BAV with an incidence of 1–2% in the general population [1,2,3]. Based on the multifactorial nature of aneurysm origin and development, the first triggers leading to aortic dilatation of sporadic forms and BAV-ATAAs are not yet known, although the aneurysmal ascending aorta is characterized by medial degeneration, a being consequence of the death of smooth muscle cells and the degeneration of elastic fibers, which together lead to progressive weakening of the wall and resulting in aortic dilatation [1,4].

As early as 2012, our group showed that pathological aortic wall changes are more pronounced in degenerative aneurysm forms associated with a TAV than in BAV-associated forms. The pathological changes mainly affected components of the extracellular matrix and characteristic properties of the smooth muscle cells [5]. Whether cell intrinsic defects or systemic processes (e.g., hemodynamics) are the first triggers for the formation of BAV-ATAA is not yet entirely clear, but the hypothesis of the “intrinsic defect” is currently favored and supported by well-founded data [6,7]. In 2002, Bauer et al. performed histological studies, showing thinner elastic lamellae in BAV-ATAAs compared to TAV-ATAAs, while no change in aortic media thickness was observed between the groups (a control group was not included in the study) [8]. One year later, Bechtel et al. reported that the histopathological changes within the aortic media are less severe in patients with a BAV than in patients with a TAV, which is remarkable due to the comparable aortic diameters in the groups [9]. In 2008, Collins et al. showed less elastic fiber-fragmentation and elastic fiber-loss in BAV-ATAAs compared to TAV-ATAAs, but BAV patients showed an impairment of elastic laminae [10]. Interestingly, Tang et al reported a reduction in media thickness, an increase in intima thickness, and an increased medial area with respect to a preserved smooth muscle cell density in small and larger aneurysms, compared to non-aneurysmal tissue [11]. In summary, these earlier studies already showed indications of a different histology in BAV- and TAV-associated forms. Apart from histological data, a recent study also showed significant differences in the metabolic profiles of BAV and TAV aneurysms [12].

Changes in smooth muscle cell phenotypes in situ have been reported, but are controversially discussed, particularly with regard to the question of whether a loss of smooth muscle cells is a dilatation-initiating or dilatation-contributing factor. In this context, Della Corte et al. could show an increased apoptotic index (only in the concavity region and depending on the degree of dilatation) in the TAV- and BAV-associated aneurysmal tissues compared to the control tissue, although the density of the smooth muscle cells in aneurysmal tissue did not change compared to the control tissue [13]. In addition, data on an increased apoptotic index in BAV-ATAAs compared to TAV-ATAAs were reported [14]. Contrary to the assumption that the loss of smooth muscle cells contributes significantly to the development of aneurysms, Tang et al. proposed a model of “dynamic medial remodeling” in which mechanical stress triggers cellular proliferation and also that the density of smooth muscle cells remains constant [11].

Although BAV-associated and TAV-associated ATAAs both have the “aneurysm” in common, the processes leading to ATAA formation must not be based on the same pathogenesis. Today, neither the disease initiating and driving forces for aneurysm development nor the consequent changes in the vascular wall are precisely defined and clarified. These circumstances still result in controversial discussions on the similarities and differences in the pathogenesis of TAV- and BAV-ATAAs.

The aim of this study was to gather new, more precise and in depth data on the structure of the aortic wall and of isolated smooth muscle cells of controls, and TAV- and BAV-ATAAs, to test the hypothesis that despite their similar macroscopic appearance, BAV-ATAA and TAV-ATAA may be based on two different pathogenic processes, and may therefore be from two different diseases, with a similar outcome.

## 2. Results

### 2.1. Study Population

The study population comprised a control group and a TAV- as well as a BAV-ATAA group. The exclusion criteria were known genetic diseases associated with aneurysm formation, patients with aortitis or dissections, aneurysm patients with an aortic diameter below 4.5 or over 6cm, and patients older than 80 years. Further exclusion criteria were known family history of aneurysms, patients with secondary acquired BAV (due to valve calcification), and patients with aortic valve replacement before surgical dilatation repair. Non-aneurysmal control ascending aortic tissues were obtained from 31 heart transplantation donors or recipients without showing diseases of the ascending aorta and characterized by a mean age of 53.3 years (±13.8 years). The study group included 47 patients with an ATAA. Based on the aortic valve morphology, the patients were divided into ATAAs associated with either a TAV or a BAV. The clinical characteristics of the individual groups are depicted in Table 1. The mean age of the TAV-ATAA group (65.7 (±11.3 years) was significantly higher compared to the control group (53.3 ± 13.8 years) as well as compared to the BAV-ATAA group (52.8 ± 13.8 years). The gender differences between the groups are obvious, as the BAV-ATAA group comprised of significantly less female patients as compared to TAV-ATAAs, as well as compared to the control group. The internal aortic diameter of the ascending aorta was determined using a cross-sectional computed tomography scan (measured from intima to intima). The internal aortic diameter, determined only in aneurysmal groups, differed significantly (TAV: 55.75 ± 3.20mm versus BAV: 53.26 ± 3.60mm) although the difference is very small (2.5mm). The tissue samples of aneurysm patients were obtained from the maximally dilated part (tissue samples of the surrounding non-aneurysmal area were not analyzed). The prevalence of smoking was generally rather low in the three study groups and statistical analyses revealed no differences between the groups. Half of the patients in the control group suffered from coronary heart disease (CHD), significantly more patients than in the BAV group and considerably more than in the TAV group. Although 75% of patients with a TAV-aneurysm suffered from hypertension, no statistically significant difference compared to the prevalence in the other two groups was detectable. There were no statistically significant differences in the prevalence of hyperlipidemia between the three groups. Interestingly, the prevalence of diabetes was significantly lower in TAV patients and showed a non-significant trend (0.053) to be lower in BAV- patients compared to the control group. Aneurysm patients with a TAV had a higher incidence of aortic stenosis compared to the controls and the BAV group. Additionally, patients suffering from an ATAA showed a significantly higher incidence for aortic regurgitation compared to the control subjects. (Table 1).

### 2.2. The TAV-ATAA Wall is Characterized by a Thinner Aortic Media, Atherosclerotic Changes, a Pronounced Media Degeneration, and Fibroblast Accumulation

**Figure 1:**
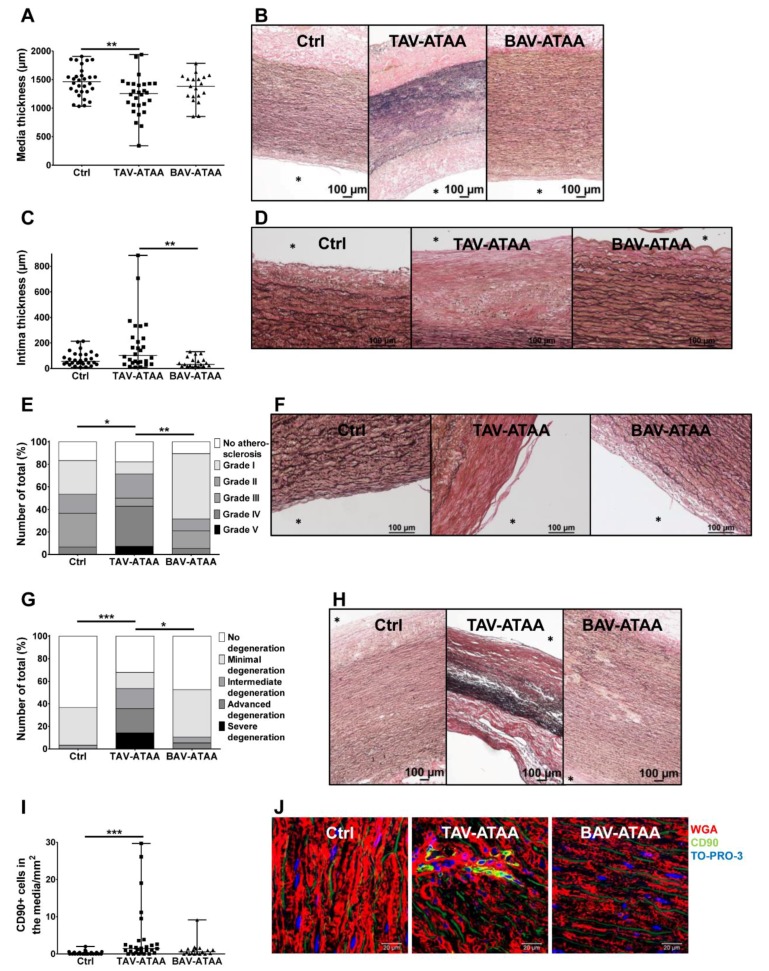
Changes in wall layer dimensions, atherosclerosis, media degeneration as well amount of fibroblasts/myofibroblasts in the aortic media of aneurysm patients and the control group. (**A**) shows individual sample values (and median/range) of media thickness of the three study groups. (**B**) shows representative images of EVG stained tissue sections for the quantification of media thickness (magnification 10×, assembled images). (**C**) shows individual sample values (and median/range) of intima thickness of the three study groups. In (**D**) representative images of EVG stained tissue (magnification 20×) for intima thickness measurements are shown. In (**E**) the extent of atherosclerosis in the aortic wall of study groups, determined by an adopted AHA grading, is shown. (**F**) shows representative images of EVG stained tissue used for the determination of atherosclerosis severity degrees (magnification 20×). In (**G**) the quantification of media degeneration extent, determined by a detailed grading system, is shown. Representative EVG stained aortic tissue sections used for media degeneration grading are depicted in (**H**) (image magnification 10×, assembled images). In (**I**) the quantification of the amount of CD90 positive cells within the aortic media is shown. (**J**) depicts representative images (magnification 63×) for anti-CD90 antibody staining (green), cell membrane staining with WGA (red) and cell nuclei staining with TO-PRO-3 (blue). (Control *n* = 30; TAV *n* = 28; BAV *n* = 19) (* < 0.05, ** < 0.01, *** < 0.001). The asterisk (*) marks the luminal side of the vessel.

The quantification of media thickness in the tissue samples revealed a significantly reduced media thickness in TAV-ATAA patients (1220.4 µm) compared to the control group (1459.7 µm) (Figure 1A,B with representative images). The media thickness in patients with a BAV (1349.4 µm) displayed no significant differences compared to TAV patients and the control group. The thickness of the intimal layer in TAV-ATAAs (180.03 µm) sections was significantly increased compared to BAV-ATAA patients (50.05 µm). No significant difference was observed when TAV-ATAAs were compared to the control group (74.5 µm) (Figure 1C; for representative images see Figure 1D). Using an adopted AHA grading system [15,16] we quantified the presence and severity of atherosclerotic aortic wall changes. The analyses revealed that the overall severity of atherosclerosis was significantly higher in TAV-ATAA patients compared to both, the control and the BAV-ATAA group. The severity of atherosclerosis did not differ between the BAV-ATAA and the control group (Figure 1E; for representative images of EVG stained section see Figure 1F). Likewise, degenerative changes within the aortic media were only present (statistically significant) in TAV-associated ATAAs compared to the control and the BAV group (for quantifications see Figure 1G; for representative images of EVG stained tissue see Figure 1H). Of note, within the ATAA population, female patients were more severely affected by atherosclerosis as well as media degeneration than male patients (Figure S1a,c). Aside from degenerative changes, immunofluorescence-based analysis revealed an increased number of fibroblasts/myofibroblasts present in the media of TAV patients compared to the control group (Figure 1I). As can be seen in the representative images, the distribution of the fibroblasts/myofibroblasts in the TAV-aneurysm samples is not even but focal (Figure 1J).

### 2.3. Focal Elastic Fiber Loss and Decreased Collagen Content in TAV-ATAAs

**Figure 2:**
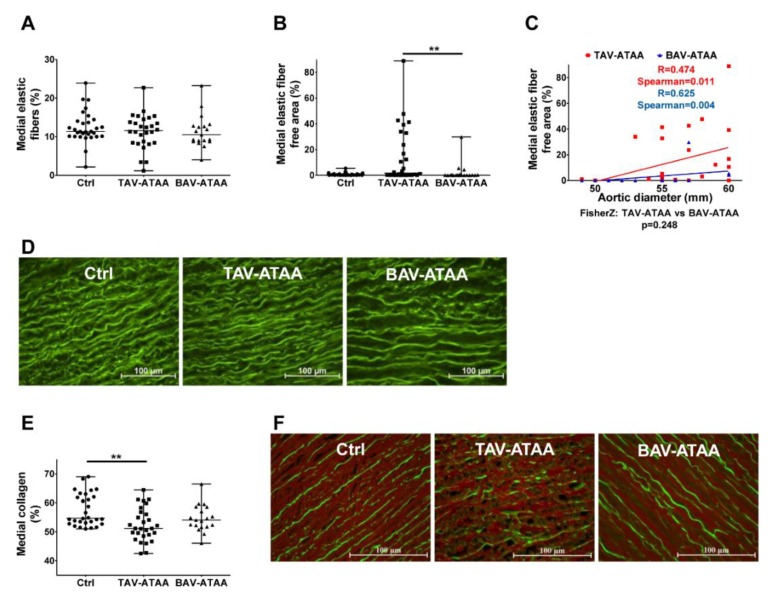
Focal degradation of elastic fibers in TAV- and BAV-associated aneurysms as well as TAV-associated loss of medial collagen amount. (**A**) shows the quantification of the content of elastic fibers relative to the total area in the aneurysmal aortas compared to the control tissue. In (**B**) the quantification of elastic fiber free areas is shown. Correlation analyses of aortic diameter with the elastic fiber free area are shown in (**C**). In (**D**) images show the auto-fluorescence properties of elastic fiber, which were used for quantification of elastic fiber content and elastic fiber free area (magnification 40×). (**E**) shows the quantification of the collagen content relative to the total area in the aneurysmal aorta compared to the control tissue (using the Sirius red stained tissue sections). (**F**) shows representative images of Sirius red stained medial tissue after fluorescence based detection of collagen (red) and viable tissue parts/elastic fibers (green) (magnification 40×). (Control *n* = 30; TAV *n* = 28; BAV *n* = 19) (** < 0.01).

Our results (Figure 2A) show no change in overall elastic fiber content, neither in TAV-ATAAs, nor in BAV-ATAAs compared to the control group. However, in TAV-ATAAs, a significant focal loss of elastic fibers was observable, compared to the BAV-ATAA study population (Figure 2B). Although the amount of elastic fiber free areas was not increased in BAV-ATAAs compared to the control group, correlation analyses revealed a significant positive correlation with the aortic diameter, present in both aneurysm groups (Figure 2C, representative images in Figure 2D). No change in elastic fiber length (overall and also not site-specific close to the adventitia, close to the intima) was observable (and lamellar units, Figure S2a–d). Quantifications of the medial collagen content revealed a significantly reduced amount of collagen in the TAV-ATAA group compared to the control, but no change in the BAV-ATAA group compared to the other two groups (Figure 2E, representative images in Figure 2F). Elastic fiber length measurements revealed the presence of shorter elastic fibers near the intima compared to the adventitial side in both aneurysmal tissue types. This difference in the length of the elastic fibers was not present in the control group (Figure S2a,b).

### 2.4. Age-Dependent Calcification and Increased OPN Expression in BAV-ATAAs

**Figure 3:**
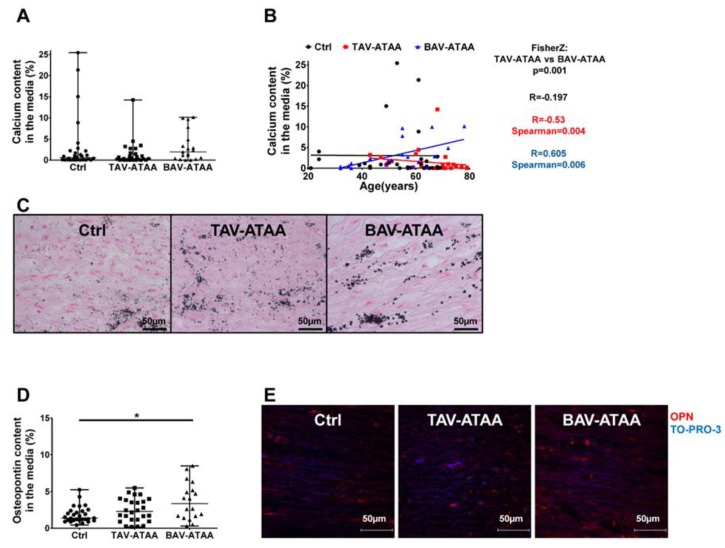
BAV-associated medial calcification and increased OPN expression. (**A**) shows the quantification of the amount of calcification relative to the total area within the aortic media of aneurysmal and control tissue. In (**B**) correlation analysis of patient age with medial calcium content is shown. In (**C**) representative images of von Kossa stained medial aortic tissue sections are shown (magnification 40×). (Control *n* = 30; TAV *n* = 28; BAV *n* = 19) Immunofluorescence-based detection and quantification of OPN expression within the aortic media is shown in (**D**). (Control *n* = 29; TAV *n* = 26; BAV *n* = 19) In (**E**) representative images of double stained tissue sections are shown: OPN specific staining is red; cell nuclei are stained in blue (TO-PRO3; magnification 60x). (* < 0.05).

Quantitative analyses showed an insignificant trend (*p*-value = 0.078) for an increased calcium content in the aortic wall of BAV patients in comparison to the other two groups (Figure 3A). Interestingly, our analyses revealed that the aortic calcium content correlates positively with patient age in BAV-ATAAs, but negatively in TAV-ATAA patients (Figure 3B; representative images in Figure 3C). Incubation of isolated smooth muscle cells from controls, TAVs, and BAVs with pro-osteogenic factors had neither an influence on calcification nor on ALP-activity (Table S2, Figure S3). *In situ*, the expression of the calcification-associated protein OPN in the aortic media was significantly increased in the BAV-ATAA samples compared to the controls (Figure 3D and representative images in Figure 3E).

### 2.5. The Increased Amount of OPN Within the Medial Layer of BAV Patients is Caused by Smooth Muscle Cells

**Figure 4:**
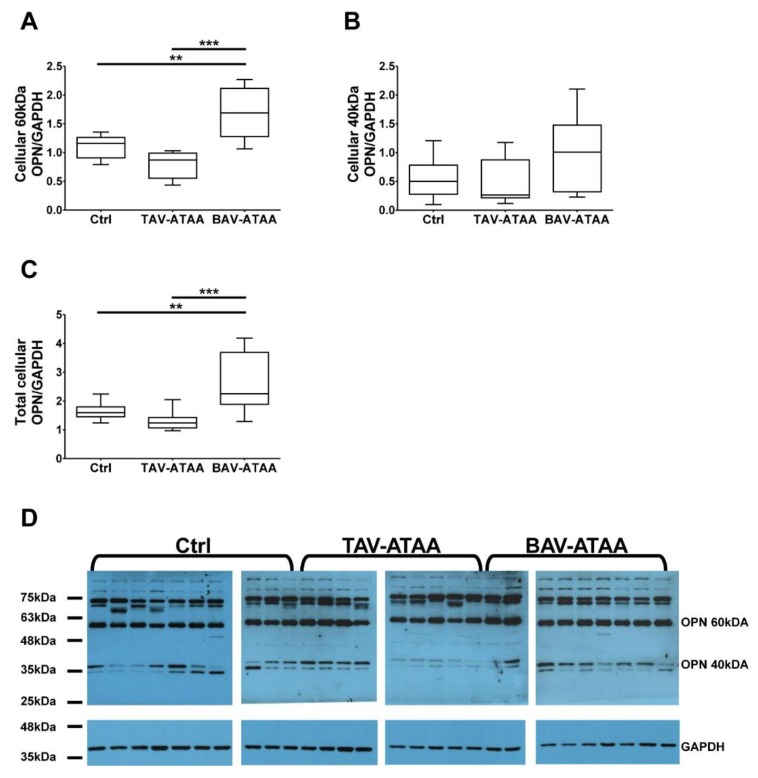
Expression pattern of OPN in isolated smooth muscle cells of aneurysmal and control tissue. (**A**) depicts the determination of uncleaved OPN expression in isolated smooth muscle cells (full length 60 kDa bands). In (**B**) the quantification of the expression of the cleaved 40 kDa band from aneurysmal and control smooth muscle cells is shown. In (**C**) the quantification of the smooth muscle specific total OPN expression is depicted (60 kDa and 40 kDa). In (**D**) the corresponding images of Western blot results for the detection of full length and cleaved OPN, as well as GAPDH (37 kDa) as loading control are shown (Control *n* = 10; TAV *n* = 10; BAV *n* = 10) (** < 0.01; *** < 0.001).

Figure 4A shows that the expression of the 60 kDa version of OPN is significantly increased in smooth muscle cells from BAV patients compared to the TAV patients and the control group, while the amount of cleaved protein increased only slightly (Figure 4B). Western Blot analyses revealed an increased total OPN expression in BAV-ATAA medial smooth muscle cells (cleaved OPN together with full length OPN) in relation to the expression of GAPDH (Figure 4C). The results suggest, that the increased OPN expression in the vascular wall of BAV patients is mainly caused by the increased expression in medial smooth muscle cells. The corresponding images of Western blot results are shown in Figure 4D.

### 2.6. Increased Smooth Muscle Cell Density and Reduced Cellular Expression of Alpha Smooth Muscle Actin in TAV-ATAAs

**Figure 5:**
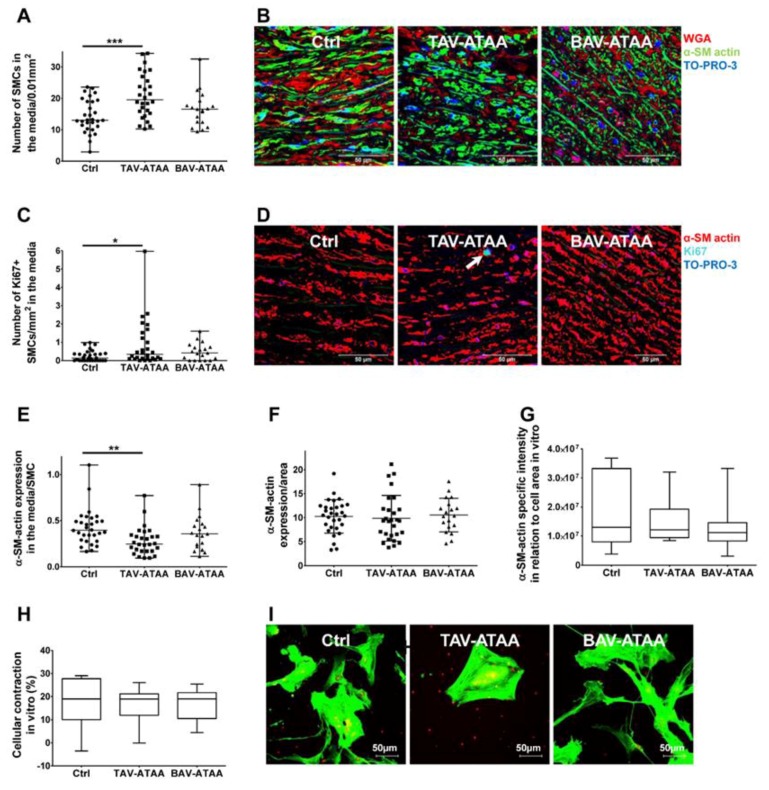
TAV-associated increase in smooth muscle cell density and decrease in α-SM actin expression per smooth muscle cell in situ. (**A**) shows the results of the quantification of smooth muscle cell density in aneurysmal and control media tissue specimens. In (**B**) representative images of aortic tissues following immunofluorescence based detection: wheat germ agglutinin (WGA, cell surface, red), α-SM actin (green), and cell nuclei (TO-PRO-3, blue) are shown. Magnification of images is 63x. In (**C**) the quantification of the amount of Ki67 positive smooth muscle cells is depicted with corresponding representative images shown in (**D**). Magnification of images taken is 63×. (**E**) shows the quantification of α-SM actin expression per smooth muscle cell within the aortic media. (Control *n* = 30; TAV *n* = 28; BAV *n* = 19) In (**F**), the quantification of α-SM actin expression per medial area is shown. (Control *n* = 30; TAV *n* = 28; BAV *n* = 19) In correspondence, (**G**) gives the quantification of the expression of α-SM actin within the isolated smooth muscle cell-lines *in vitro*. (**H**) shows the results of the quantification of smooth muscle cell-contraction (% of the length of relaxed cells in relation to cell area) ability in response to carbamylcholine chloride in vitro. (Control *n* = 9; TAV *n* = 10; BAV *n* = 10) (**I**) shows representative images of isolated smooth muscle cells stained for α-SM actin (magnification 60×). (** < 0.01).

A quantification of the number of smooth muscle cells per area revealed an increased smooth muscle cell density in the aortic media of TAV-ATAA patients compared to the control group (Figure 5A, Figure S5a). Of note, statistical evaluations showed that this parameter is positively correlated to the age of the patients (Table S5). Differences in the number of smooth muscle cells and α-SM expression between the media near intima side and the media near adventitia side are summarized in Figure S4. Figure 5B depicts the representative images of tissue sections stained with anti-α-SM actin antibody (green), wheat germ agglutinin (WGA) (red) for the cell surface, and TO-PRO-3 (blue) for cell nuclei staining. Fluorescence based staining revealed a significant increase in the number of Ki67 positive smooth muscle cells in the media of TAV-ATAA specimens compared to the control tissue (Figure 5C, representative images in Figure 5D show an even distribution of positive cells in the media). Surprisingly, in spite of the increased density of smooth muscle cells, these smooth muscle cells had a significantly lower expression of α-SM actin per cell compared to the controls (Figure 5E). The quantification of α-SM actin expression per area indicate that there is no significant difference between TAV patient samples and the two other groups (BAVs and controls) (Figure 5F). Isolated TAV patient smooth muscle cells in vitro did not show the reduced cellular α-SM actin expression phenotype that was observed in situ (Figure 5G, representative images in Figure 5I). Similarly, the isolated smooth muscle cells from TAV patients showed no change in their contractile properties (in relation to the cell area), as shown in Figure 5H. Neither PDGF-BB nor FGF-1 expression seem to be responsible for proliferation of smooth muscle cells (Figure S5a–d).

## 3. Discussion

Although essential differences in the pathogenesis between BAV and TAV associated aneurysms have been reported [17,18,19,20,21,22], recently published data are still controversial on this issue. The final proof of the concept, that the BAV- and the TAV-ATAA are two different disease entities is still missing and the specific processes of ATAA formation in patients with BAVs (as reviewed in [23]) and TAVs are still far from understood. Formation of well-matched study populations is difficult when comparing TAV with BAV patients, as TAV patients on average are much older than BAV patients on the day of surgery. In addition, there are often significant differences in aneurysm diameters, as BAV patients generally undergo surgery at lower diameters than TAV patients. Even more, the indicator for surgery, i.e., the aortic diameter, differs from center to center.

The results from detailed histological and immunofluorescence based analyses of aortic tissue revealed pronounced and strong changes, which indicate a considerable weakening of the aortic wall in TAV patients. These changes affect the thickness of the layers as well as the structural and cellular components. Despite significant efforts in the field, the present study reveals new and so far unknown alterations in TAV patients’ aorta. The aortic wall layer dimensions are strongly shifted in TAV compared to BAV patients and controls. The increased intima thickness (as already described [11]) together with the occurrence of massive atherosclerotic changes provides clear evidence for a tight association of TAV associated aneurysms with atherosclerosis (also see Albini et al. [15]). Another issue in ATAA research that is still controversial, as already discussed in the following publications [24,25]. The reduced thickness of the media together with the strongly pronounced media degeneration is an expression of a further weakening of the aortic wall in TAV patients. This media degeneration, already described for aneurysms [1,4], is accompanied by an appearance of fibroblasts (or myofibroblasts, as already described in a murine aneurysm model [26]). This shift in cell composition may indicate prior tissue damage and may further affect the stability of the aortic wall, since fibroblasts or myofibroblasts cannot replace contractile smooth muscle cell function. In contrast to most of the reports published in the recent decades [1,3,11,20,27], our analyses gave that the aortic media of both aneurysm groups had an unchanged total elastic fiber area compared to the control group. Our new data suggest, that media degeneration occurs i) focally but ii) without changing the total amount of elastic fibers. In contrast to previous data [28], we could not observe even the smallest signs of media degeneration in BAV-associated ATAAs.

The data on the collagen content in aneurysmal tissue reported in prior work ranges from an increased, to a constant, to a decreased amount in ATAAs (summarized by Tsamis et al. [29]). We and others [11,23,24] observed a disease-progression-related decrease in the medial collagen content in TAV-ATAAs only. Taking into account previous studies showing that disrupted aortic tissue contains less collagen after aneurysm rupture or dissection [29], the pre-existing reduced amount of collagen makes the tissue more prone to rupture and dissection. Collagen recruits mainly to sites of high pressure peaks and this recruitment may be impaired in aneurysm tissue.

The pathological changes on the cellular level in TAV-ATAAs not only mean the presence of fibroblast/myofibroblasts in the aortic media, but also significant alterations in the most mechanically important cell type in aortic media, the smooth muscle cell. As already suggested by Tang et al. [11] in their "model of dynamic medial remodeling" however in contrast to other studies (e.g., Della Corte et al. [13]) we are the first to provide clear evidence for the increase in smooth muscle cell density in the media of TAV-ATAAs. This observed change in smooth muscle cell density is likely to reflect a pathophysiological relevant adaptive compensation process. This view is even more supported by the fact that smooth muscle cells in TAV patients express significantly less α-SM actin per cell than controls. Interestingly, even though the α-SM actin expression per cell is reduced, due to the increased smooth muscle cell density the overall amount of α-SM actin in the media remains constant between the groups. The fact that in vivo findings on smooth muscle cell density and α-SM actin per cell are not seen in in vitro culture isolates may argue for a role of biomechanical factors in the in vivo phenotype, rather than a pure intrinsic problem of the TAV-ATAA smooth muscle cell.

Despite the fact that patients with a BAV develop an aneurysm of comparable size to that of TAV patients, far less pathological wall changes are associated with BAV aneurysm development. However, a trend towards an increased calcification of the aortic wall in BAV patients, which is also strongly associated with patient age within the BAV group may argue for a role of calcification in BAVs and may be specific for BAV-ATAA formation. Medial calcification is per definition an age-related degenerative process [30] which, according to current knowledge, is associated with undesirable cardiovascular events such as aortic dissection and rupture [31]. Since BAV patients are significantly younger compared to TAV patients in this analysis, the process of calcification is perhaps aneurysm-associated rather than age-associated. It is noteworthy that signs for early and accelerated calcification and the presence of pro-osteogenic signals are already reported for the bicuspid valve [32], but we are the first to report such results for the BAV aortic wall. Medial calcification is complex and a not fully understood process, generally the induction of OPN is hypothesized to inhibit vascular calcification by preventing apatite crystal growth [33,34,35]. Further it has also been shown that smooth muscle cells that no longer produce OPN are much more susceptible to calcification [36,37]. The data presented herein suggest however, that the situation in the aorta of BAV patients may be different, since despite significantly increased expression of OPN by smooth muscle cells, increased calcification of the aortic wall is evident. Thus, OPN may have a dual role. Clearly, in this study we do not report on the functional relationships between OPN and calcification in BAV aneurysm. Accordingly, the “dual role of OPN in calcification” remains a hypothesis.

## 4. Material and Methods

### 4.1. Study Population

Tissues used in the present study were collected at two Austrian Universities in order to increase group size. The study is approved by the respective ethics committees of the Medical University of Vienna (1183/2012) and the Medical University of Innsbruck (EK1 09.11.12). For further details regarding the study population please see Table 1 in the results section.

### 4.2. Histological Stainings and Analyses

Aortic tissues were fixed in 4.5% buffered formaldehyde solution, dehydrated (35 min in 100% EtOH, 70 min in isopropyl alcohol, and 90 min in paraffin) using a KOS Microwave Histostation (Milestone, Sorisole, Italy/BG), and subsequently embedded in paraffin. After preparation of 5 µm thick sections and deparaffinization of them (2 × 10 min in HistoSAV, 2 × 5 min in 100% EtOH, 2 × 5 min in 96% EtOH, 1 × 2 min in 80% EtOH, 1 × 2 min in 70% EtOH, 1 × 2 min in 50% EtOH, and 1 × 2 min in A.d.), staining of elastic fibers (Elastica van Gieson, EVG; Merck Millipore, Darmstadt, Germany), collagen (Sirius Red, SR; Merck Millipore, Darmstadt, Germany), and calcium depositions (Silver staining to van Kossa; Merck Millipore; Darmstadt, Germany) were conducted according to the manufacturer’s instructions, and the slides were subsequently mounted using Eukitt (Merck Millipore, Darmstadt, Germany). Image acquisitions were performed using Zeiss AxioVision microscope (Carl Zeiss, Oberkochen, Germany) and connected 4.8.2 software (Carl Zeiss, Oberkochen, Germany). EVG stained tissues were used to quantify intima and media thickness (expressed in µm). In detail, 10–30 measurements at 2–4 different positions across the aorta were used to determine the intima (magnification of images 20×) as well as media thickness (magnification of images 10×). Additionally, EVG stained tissues were used to quantify atherosclerotic changes (using images of whole aortic tissue). For this purpose we used a modified AHA classification system and established a classification consisting of 5 grades (grade 0 to grade 4)—grade 0 = no thickening of the intimal layer; grade I = clearly visible intimal thickening (4–8 cell layers); grade II = intimal thickening containing isolated foam cells; grade III = more than 20 intimal cell layers containing several foam cells, and leukocyte infiltrations; grade IV = pronounced intimal thickening, massive foam cell infiltration, and beginning deposition of cholesterol crystals. Likewise, EVG stained tissues were used to determine the grade of media degeneration in aortic specimens according to the classification published by Roberts et al. [38] The following grades were used to classify the aortic specimens—grade 0 implies tissues without visible signs of media degeneration; grade 1 represents tissue with breaks and loss of isolated elastic fibers (minimal fragmentation); grade 2 specimens showed small but distinct areas without or with only fragmented elastic fibers (intermediate fragmentation); grade 3 represents tissue with significant loss of elastic fibers in several areas (advanced fragmentation); grade 4 samples showed complete loss of elastic fibers in large areas of the media (severe fragmentation). The collagen content was determined using Sirius red stained tissue sections and a Nikon Eclipse Ti microscope (Nikon Instruments, Minato, Tokio, Japan). Image analysis was performed using the NIS elements BR 4.20.01 software (Nikon Instruments, Minato, Tokio, Japan) as described by Vogel et al. [39]. The stained tissue sections were investigated under fluorescence light (460 nm) at a magnification of 40× and the collagen content was then calculated as the red fluorescent area compared to the total area. The overall medial calcium depositions were analyzed using Adobe Photoshop (CS5, San Jose, CA, USA) and quantified as a positive area (dark brown to black) to the total area. The side specific differences in calcium deposition were analyzed by dividing the media into two equal parts. Images were taken at the side near the intima and at the side near the adventitia at a magnification of 40×. The analyses were performed by blinded researchers.

### 4.3. Auto-Fluorescence Based Analyses and Quantifications

Using the auto-fluorescence properties of elastic fibers and aortic collagen, we quantified the elastic fiber content, the elastic fiber thickness, and the elastic fiber length on 3 µm thick histological sections after rehydration, antigen retrieval in Tris/EDTA buffer (10 mM Tris, 1 mM EDTA (Ethylenediaminetetraacetic acid), pH 9) using a KOS Microwave Histostation (15 min at 98 °C; Milstone, Sorisole, Italy/BG), and mounting of slides using ProLong Gold Antifade (Thermo Fisher; Vienna, Austria). Image acquisition (magnification 40×) was performed using a Nikon Eclipse Ti microscope (Nikon Instruments, Minato, Tokio, Japan) and image analysis was conducted using NIS elements BR 4.20.01 software (Nikon Instruments, Minato, Tokio, Japan). All examinations were performed at six different positions within the media of the aortic tissue sections. Quantification of the stronger autofluorescence, attributable to the elastic fibers, was performed and calculated as an area in regard to the total area (%). The stronger autofluorescence of elastic fibers was also used to measure the fiber thickness of at least 15 to 20 fibers and the length of at least 10 to 15 fibers per investigation area expressed in µm. The analyses were performed by blinded researchers.

### 4.4. Immunofluorescence Based Stainings and Quantifications

After fixation, embedding in paraffin, and cutting, the 3 µm aortic tissue sections were re-hydrated and antigen retrieval was performed according to the suggestions of antibody manufacturers. After washing with TBS (Tris buffered saline; 3 times for 5 min), blocking of unspecific binding sides with 10% goat serum/1% bovine serum albumin (BSA; Merck Millipore, Darmstadt, Germany) in TBS (Tris-buffered saline) was performed. Staining of fibroblasts/myofibroblasts within the aortic media was performed using an anti-CD90 antibody (1:150; ab92574; Abcam, Cambridge, UK). Following the incubation overnight at 4 °C, the slides were washed 3 times for 5 min with TBS/0.1% Tween 20 (Biorad, Vienna, Austria) and incubated with the appropriate goat anti-rabbit Alexa 488 secondary antibody (1:1000; A1103, Thermo Fisher, Vienna, Austria) for 60 min at room temperature. After an additional washing step, plasma membranes were stained using wheat germ agglutinin (WGA) Alexa 555 (1:200, Life Technologies, W32464) and cell nuclei were stained using TO-PRO-3 (1:1000, T3605; Thermo Fisher, Vienna, Austria). Similarly, the staining of smooth muscle cells was performed. However, before specific staining, plasma membranes of smooth muscle cells were stained with WGA Alexa 555 (1:200, W32464; Thermo Fisher, Vienna, Austria). Staining of smooth muscle cells was performed after permeabilisation of plasma membranes with 0.2% Triton X-100 in PBS (Phosphate-buffered saline), using a rabbit polyclonal anti-α-SM actin antibody (1:150, ab66133; Abcam, Cambridge, UK) and the appropriate secondary goat anti-rabbit Alexa 488 antibody (1:1000, A11034; Thermo Fisher, Vienna, Austria). Likewise, the cell nuclei were stained with TO-PRO-3 (1:1000, T3605; Thermo Fisher, Vienna, Austria). In order to analyze the proliferative activity of aortic smooth muscle cells, tissues were permeabilized as described above and double stained with mouse monoclonal anti-α-SM actin antibody 1:400 (used dilution according to manufacturer’s instructions, ascites fluid, A2547 Clone 1A4; Merck Millipore, Darmstadt, Germany) and rabbit polyclonal anti-Ki67 antibody (1:500, ab15580; Abcam, Cambridge, UK). Secondary antibodies used were goat anti-mouse Alexa 546 (1:1000; A11030, Thermo Fisher, Vienna, Austria) and goat anti-rabbit Alexa 488 (1:1000; A11034, Thermo Fisher, Vienna, Austria) respectively. To stain the cell nuclei, TO-PRO-3 (1:1000; T3605, Thermo Fisher, Vienna, Austria) was used. Image acquisition was performed using a Zeiss LSM 500 laser confocal microscope and Zen 2008 software (Version 5.0; Carl Zeiss, Oberkochen, Germany). The number of fibroblasts/myofibroblasts and smooth muscle cells was analyzed by counting CD90+ cells and α-SM actin + cells per mm^2^. Quantification of α-SM actin per cell was performed by quantification of α-SM actin area in relation to the number of smooth muscle cells per field of investigation. The number of proliferative cells was quantified by counting the number of Ki67+ smooth muscle cells within the aortic media. Staining against OPN (1:300, used dilution according to manufacturer’s instructions, ascites fluid; ab8448, Abcam, Cambridge, UK) was performed as described above for the Ki67 staining, but without prior permeabilisation and α-SM actin staining and with secondary goat anti-mouse Alexa 546 (1:1000; A11030; Thermo Fisher, Vienna, Austria). All the antibodies used have been diluted according to the manufacturer’s instructions and the application used. Finally, all the stained tissue sections were mounted with ProLong Gold antifade (Thermo Fisher, Vienna, Austria). The OPN content was determined by quantifying the percent positive area in relation to the whole area. Image acquisition was performed using a Nikon Eclipse Ti microscope using NIS Elements software (Nikon Instruments, Minato, Tokio, Japan). The analyses were performed by blinded researchers.

### 4.5. Isolation of Primary Smooth Muscle Cells

Primary smooth muscle cells were isolated from human aortic tissue as shortly as possible after surgery. Aortic tissue was washed three times in PBS-/- (without CaCl_2_ and MgCl_2;_ Thermo Fisher, Vienna, Austria). Afterwards the tunica adventitia was separated to avoid fibroblast contamination and the endothelial cells were scratched off using a scalpel. Subsequently, the media was washed again three times in PBS-/- and cut into small pieces followed by incubation in an enzyme mixture (147.5 U/mL collagenase Type IV [Gibco, Thermo Fisher, Vienna, Austria] and 5 U/mL pancreatic elastase [Calbiochem, Merck Millipore, Darmstadt, Germany]) for 3 h at 37 °C. Afterwards, the cells were separated from residual tissue using a cell strainer (100 µm) and collected by a centrifugation step (300 g, 10 min). Finally, the cell pellet was re-suspended in specific smooth muscle cell media (Medium 231, Cascade Biologics, Thermo Fisher, Vienna, Austria) containing smooth muscle cell specific supplements (SMGS, Cascade Biologics, Thermo Fisher, Vienna, Austria).

### 4.6. Cell Contraction Assay

The contractility of isolated smooth muscle cells was analyzed by incubating cells with 10^−4^ M carbamoylcholine chloride (MedChem Express, NJ, USA) for 30 s, to induce contraction followed by fixation with acrolein (Merck Millipore, Darmstadt, Germany) to a final concentration of 1%. The cells were washed 3 times using PBS-/- and afterwards stained with 5 U/mL Alexa Fluor 488 Phalloidin (Thermo Fisher, Vienna, Austria) in PBS with 1% BSA for 30 min. Subsequently cells were washed 3 times using PBS-/- and the cell nuclei were stained with DAPI (Thermo Fisher, Vienna, Austria). By measuring the cell length of untreated versus cells treated with carbamoylcholine, the contraction was calculated as percent. DAPI staining was used to distinguish between cells and debris. The analyses were performed by blinded researchers.

### 4.7. Immunofluorescence-Based Staining of Primary Smooth Muscle Cells

Primary smooth muscle cells were seeded, and fixed by 4% paraformaldehyde for 5 min. After washing with PBS-/- (3 times for 5 min), permeabilization with 0.3% Triton-X for 30 min, additional washing step, and blocking with 1% BSA for 30 min were performed. Incubation using primary mouse monoclonal anti-α-SM actin antibody (A 2547; Merck Millipore, Darmstadt, Germany) with a dilution of 1:400 was performed overnight at 4 °C. The next day, after a washing step, the incubation with the secondary goat anti-mouse Alexa 488 antibody (A11029, Thermo Fisher, Vienna, Austria) was performed with a dilution of 1:1000 for 1 h at room temperature. Subsequently an additional washing step and nuclei staining using propidium iodide (500 ng/µL) for 1.5 min and a final washing step was performed, followed by the mounting of the sections with ProLong Gold antifade (Thermo Fisher, Vienna, Austria). Image acquisition was performed using a Nikon Eclipse Ti microscope and NIS Elements software (Nikon Instruments, Minato, Tokyo, Japan). The analyses were performed by blinded researchers. The α-SM actin amount was determined by calculating the expression intensity in relation to the cell area.

### 4.8. Western Blot Analyses

The isolation of proteins from primary smooth muscle cells was performed as described previously [40]. The detection of specific protein expression was performed using antibodies against OPN (1:2000; 25715-1-AP, Proteintech, UK), p27 (1:500; 610242, BD Bioscience, Vienna Austria), cyclin D1 (1:400; ab16683, Abcam, Cambridge, UK), and GAPDH (1:2000; 2118L Cell signaling, Germany). The quantification of protein bands was performed using the program ImageJ (NIH, version 1.51d) and the expression profile was calculated in relation to the expression of the house keeping protein GAPDH. The analyses were performed by blinded researchers.

### 4.9. Statistical Analyses

Statistical analyses were performed using SPSS 20.0 software performed by a professional statistician from the Medical University of Vienna. For comparison of the atherosclerotic and media degeneration grades as well as gender, smoking, coronary heart disease (CHD), hypertension, hyperlipidemia, diabetes, and aortic stenosis between the groups (control, TAV-ATAA, BAV-ATAA), a Chi square test or, if appropriate due to a small sample size, a Fisher-exact test was performed. To compare the continuous variables, one-factorial ANOVA with a factor group was performed and data is summarized in Supplementary Table S1. Comparison of results of adventitia-near intima with intima-near medial layer analyses are summarized in Table S3. To investigate the correlations of the analyzed parameters with age as well as aortic diameter, Spearman correlation coefficients and the corresponding tests were performed. To investigate the difference of the Spearman correlation coefficients between the groups, Fishers *z*-test was used and the results are summarized in Supplementary Table S4 (Supplementary Material). The statistical analyses comparing the patient groups of an age below 50 years (<50a) and above 50 years (>50a) are summarized in Table S2. As a second analysis, for each outcome variable, an ANOVA test was performed accounting for age (or aortic diameter), group, and the interaction between age and group. The median and range are depicted as dot plots for graphs based on histological assays; box plots with range are depicted for graphs based on primary cell culture assays. Due to the exploratory character of the study, no correction for multiplicity was performed. Further, an ANCOVA-test was used to analyze the influence of age and CHD prevalence on the results. All *p*-values below 0.05 were considered as statistically significant. For all detailed statistical analyses, please see the Supplementary Material.

## 5. Conclusions

**Figure 6:**
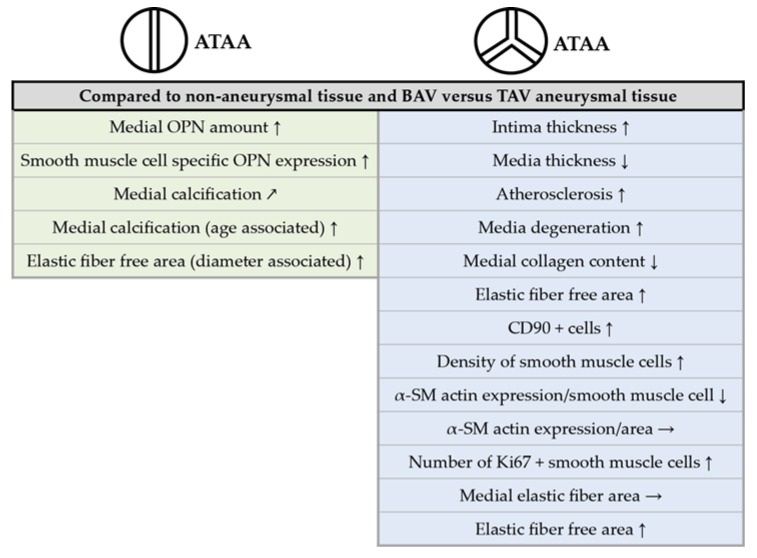
Summary of observed changes in the aortic wall of BAV- and TAV-associated aneurysm patients. Figure 6 gives an overview on classical changes in the aneurysmal aortic wall and provides new information on BAV- and TAV-ATAA phenotypes. The summary of the results indicates a massive difference between BAV- and TAV-ATAA tissues, arguing for different pathological processes to occur in the two patient groups. The TAV is characterized by massive structural changes that are likely to weaken the aortic wall; atherosclerosis as a risk factor for TAV-associated ATAA formation may be re-considered. Pathological changes in BAV-ATAA tissue, that were observed, are limited to age-associated increased calcification and increased expression of OPN in the media. The role of these phenotypes in BAV-ATAA formation remain unclear (↑ = increased; ↗ = trend to be increased; ↓ = decreased; → = unchanged).

It was the goal of the present project to gather new data to test the hypothesis that the TAV and BAV rely on two independent pathological processes that lead to a similar outcome, i.e., the ATAA. In fact, we show new results and confirm previous results which are in further support of the hypothesis that the BAV-ATAA and the TAV-ATAA are two independent diseases. The TAV associated aneurysm is characterized by a pronounced aortic wall degeneration suggesting strong wall weakening, whereas BAV aneurysm associated wall changes are limited to a tendency towards increased calcification and an increased expression of OPN. For an overview on the detected pathological changes and differences between TAV- and BAV- ATAAs and the corresponding control group, see Figure 6.

## 6. Study Limitations

In general, the results of the present study are descriptive and non-functional in nature, which only allows for the formulation of new hypotheses. The minor histological changes in our BAV study population were very surprising, particularly as the extent of dilatation in the BAV patients was very similar as in the TAV group. Although we did our best to adjust the groups, the BAV group is still significantly younger than the TAV and the control group. Nevertheless, extensive statistical testing gave that the only parameter influenced by age was the medial density of smooth muscle cells, over the entire study population. Massive structural changes postulated so far for the aorta of BAV patients are not present in our study group. The differences to other previous results are unclear. Importantly, the present study relies on strict quantitative image analyses and data and the number of samples per groups is high.

## Figures and Tables

**Table 1:** Clinical characteristics of controls and aneurysm patients.

						*p*-Value		
			C	T	B	C vs. T	C vs. B	T vs. B
Number			*n* = 30	*n* = 28	*n* = 19			
Age (years)			54.4 ± 12.8	65.7 ± 11.3	52.8 ± 14	0.002	ns	0.002
Gender (male)			66.7%	57.1%	94.7%	ns	0.037	0.004
Internal aortic diameter (mm)			nd	55.8 ± 3.2	53.3 ± 3.6	nd	nd	0.033
Current smoker			6.7%	7.1%	0%	ns	ns	ns
CHD			50%	25%	15.8%	0.062	0.018	ns
Hypertension			46.7%	75%	52.6%	ns	ns	ns
Hyperlipidemia			30%	35.7%	31.6%	ns	ns	ns
Diabetes			36.7%	3.6%	10.5%	0.003	0.053	ns
Aortic stenosis			0%	15.4%	73.3%	0.037	<0.001	<0.001
			**Aortic Regurgitation**			***p*-Value**		
	No	Minimal	Mild	Moderate	Severe	C vs. T	C vs. B	T vs. B
C	18	5	2	1	0	<0.001	<0.001	ns
T	4	3	4	7	8			
B	1	2	1	7	4			

ns = not significant; nd = not determined. Control (C) *n* = 26, TAV-ATAA (T) *n* = 26, BAV-ATAA (B) *n* = 15.

## Supplementary Materials

Supplementary materials can be found at https://www.mdpi.com/1422-0067/20/19/4782/s1.
