# Supplementary material for: Strong Signs for a Weak Wall in Tricuspid Aortic Valve Associated Aneurysms and a Role for Osteopontin in Bicuspid Aortic Valve Associated Aneurysms
Source: Int J Mol Sci. 2019 Sep 26;20(19):4782. doi: 10.3390/ijms20194782 (PMC6802355; doi:10.3390/ijms20194782)
Supplement: Supplementary file 1 [file ijms-20-04782-s001.pdf]

ONLINE DATA SUPPLEMENT

Strong Signs for a Weak Wall in Tricuspid Aortic Valve Associated Aneurysms and a Role for Osteopontin in Bicuspid Aortic Valve Associated Aneurysms

1. MATERIAL AND METHODS

1.1 Immunofluorescence based stainings and quantifications of PDGF-BB and FGF-1

After fixation and embedding in paraffin, 3μm aortic tissue sections were re-hydrated and antigen retrieval was performed according to the suggestions of antibody manufacturers. After washing with TBS (Tris buffered saline) (3 times for 5 min), blocking of unspecific binding sides with 10% goat serum/1% bovine serum albumin (BSA) (Merck, Germany) in TBS. Staining against PDGF-BB (1:200, bs-0185R, Bioss, Germany) and FGF-1 (1:200, sc-1884, Santa Cruz Biotechnology, Dallas, Texas, USA) were performed as like the Ki67 staining described in the manuscript, but without prior permeabilisation and α-SM actin staining and with secondary goat anti-mouse Alexa 546 (1:1000, A11030, Thermo Fisher, Vienna, Austria) and for FGF-1 as secondary antibody donkey anti-goat Alexa 555 (1:1000, ab150130, Cambridge, USA) was used. All antibodies used have been diluted according to the manufacturer's instructions and the application used. Finally, all the stained tissue sections were mounted with ProLong Gold antifade (A11030, Thermo Fisher, Vienna,

Austria). PDGF-BB and FGF-1 expressions were determined by quantifying the percent positive area in relation to the whole area. Image acquisition was performed using a Nikon Eclipse Ti microscope using NIS Elements software. Analyses were performed by blinded researchers.

1.2 TUNEL assay

The TUNEL assay was performed on histological sections according to the manufacturer's instructions (In Situ Cell Death Detection Kit, POD; Roche Germany). Dead cells were counted as percentage of positive cells in relation to the total cell number. Analyses were performed by blinded researchers.

1.3 Cell confluence assay

Primary smooth muscle cells were seeded and layer confluence was measured over a time period of 96 hours using a Tecan Spark 20M Te-Cool (Tecan Trading, Switzerland) to determine the proliferation capacity of the cells.

1.4 Alkaline phosphatase assay

ALP assay was performed using extracts from primary smooth muscle cells and according to manufacturer's instruction (Alkaline Phosphatase Activity Colorimetric Assay Kit, BioVision, USA). Analyses were performed by blinded researchers.

1.5 In vitro Calcification assay of primary smooth muscle cells

$5x10^4$ primary smooth muscle cells were seeded in a 12-well plate and incubated with calcification media (DMEM [Lonza, Szabo Scandic Austria] +2.5mM NaH2PO4 + 4mM

CaCl2) which was exchanged every 48 hours for 7 days. Subsequently cells were fixed by adding 7.5% buffered formaldehyde solution to a 1:1 solution for 15 minutes, discarding solution and again incubating 15 minutes with pure 7.5% buffered formaldehyde solution. Afterwards buffered formaldehyde solution was removed, cells washed with distilled water and von Kossa stain was performed according to manufacturer's instruction.

2. RESULTS

2.1 Summary of all statistical analyses performed

Statistically calculated p-values testing null hypothesis of experimental analyses performed in this study are summarized in Table S1. The data obtained from cellular assays were analyzed by grouping patients in two groups (patients over 50 years of age and below) and the results are presented in Table S2. Comparisons of side specific differences (media near intima compared to media near adventitia) in the expression pattern of target parameters are summarized in Table S3. P-values below 0.05 are defined as statistically significant.

Table S1: Summary of statistical analyses performed with data obtained in the present study:

(* <0.05, ** <0.01, *** <0.001, ns = not significant)

Analyses	ANOVA p-value	Post-hoc		
		Ctrl vs TAV-	Ctrl vs BAV-	TAV- vs
**Media thickness**	**0.009**	**0.007****	ns	ns
**Intima thickness**	**0.007**	ns	ns	**0.005****

**Wall thickness**	ns	-	-	-
**Media degeneration**	**0.002**	**<0.001***	ns	**0.037***
**CD90+ cells in the**	**<0.001**	**<0.001***	0.099	ns
**Medial EF content**	ns	-	-	-
**EF free area**	**0.01**	ns	ns	**0.007****
**EF length**	ns	-	-	-
**EF thickness**	ns	-	-	-
**Lamellar units**	**0.011**	**0.011***	ns	ns
**LU/media thickness**	ns	-	-	-
**Medial collagen content**	**0.03**	**0.02***	-	-
**Atherosclerosis**	**0.003**	**0.011***	ns	**0.006****
**Calcium content in the**	0.078	-	-	-
**OPN content in the media**	**0.024**	ns	**0.019***	ns
**Cellular ALP activity**	ns	-	-	-
**Total cellular OPN/GAPDH**	ns	-	-	-
**Cleaved cellular/total OPN**	ns	-	-	-
**Cellular calcium content/nuclei**	ns	-	-	-
**Smooth muscle cellss in the**	**<0.001**	**<0.001***	ns	0.064
**Ki67+ smooth muscle cellsin the media/mm2**	**0.034**	**0.038***	ns	ns
**PDGF-BB content in the media**	ns	-	-	-
**FGF-1 content in the media**	ns	-	-	-

**TUNEL+ cells in the media/1000 cells**	ns	-	-	-
**Death cells in culture**	ns	-	-	-
**Cellular Δ confluence**	ns	-	-	-
**Cellular p27/GAPDH**	ns	-	-	-
**Cellular CyclinD1/GAPDH**	ns	-	-	-
**α-SM-actin in the media/smooth muscle cells**	**0.003**	**0.002****	ns	ns
**α-SM-actin amount in vitro**	ns	-	-	-
**Cellular contraction in vitro**	ns	-	-	-

Table S2: Summary of statistical analyses performed to evaluate differences in an age dependent clustering of groups: (* <0.05, ns = not significant)

	<50a vs >50a			
Analyses	**Overall**	**Ctrl**	**TAV-ATAA**	**BAV-ATAA**
**Cellular ALP activity**	ns	ns	ns	ns
**Cellular cleaved/total OPN**	**0.061**	ns	ns	**0.016***
**Cellular calcium content/nuclei**	ns	ns	ns	ns
**Death cells in culture**	ns	ns	ns	ns
**Cellular Δ confluence**	ns	ns	ns	ns
**Cellular p27/GAPDH**	ns	ns	ns	ns
**Cellular CyclinD1/GAPDH**	ns	ns	ns	ns
**α-SM-actin amount in vitro**	ns	ns	ns	ns
**Cellular contraction in vitro**	ns	ns	ns	ns

Table S3: Summary of statistical evaluations for side specific analyses: p-values correspond to comparison in a group between near intima and near adventitia (* = 0.05, *** = 0.001 in Ctrl group; # = 0.05, ## = 0.01 in TAV-ATAA; $ = 0.05, $$ = 0.01 in BAV ATAA)

Analyses	Near intima [absolute			Near adventitia [absolute			
	Ctrl	TAV-	BAV-	Ctrl	TAV-	BAV-	Statistical
**Medial EF content**	11.89	10.42	10.16	12.86	11.68	12.54	*, $
**EF length (μm)**	22.23	19.16	18.72	23.17	21.89	22.36	#, $
**EF thickness (μm)**	2.97	2.90	2.93	2.88	2.83	2.88	
**Medial collagen**	57.57	52.91	54.80	57.62	51.87	54.55	
**Calcium content**	1.69	0.86	1.43	3.05	1.41	3.20	***, ##, $$
**OPN content in the media (%)**	1.67	2.37	3.15	1.97	2.55	4.25	
**Smoot muscle cells in the media/0.01mm²**	11.81	18.87	16.69	16.92	22.89	16.60	***, #
**TUNEL+ cells in the**	3.00	1.30	1.46	2.57	0.67	1.63	
**α-SM actin in the media/Smooth muscle cell**	0.41	0.29	0.34	0.44	0.26	0.40	$

2.2 Correlation analyses of histological data and immunofluorescence based detections with patient age and aortic diameter.

Correlation analyses of all data from histological analyses and the expression patterns determined using immunofluorescence-based methods (except collagen content which correlates with aortic diameter and calcium content and OPN content which correlate

with patient age, which are described in the main article) are summarized in Table S4. The spearman correlation analyses revealed a significant positive correlation of media thickness with intima thickness in the control group and negative correlation in patients with a TAV. Patients with a TAV and the control group showed a significant positive correlation of LU number, media thickness, and PDGF-BB expression with patient age. In addition, patients with a TAV showed significantly positive correlation of EF free area and OPN content with the diameter as well as negative correlation of FGF-1 content with aortic diameter. Patients with a BAV showed a significantly positive correlation of EF free area with aortic diameter.

Table S4: Correlation analyses of experimental data to age and aortic diameter using Spearman and Fisher-Z-test: (* <0.05, ** <0.01, nd = not determined)

Correlations		Spearman		Fisher Z		
		R	p value	Ctrl vs	Ctrl vs	TAV- vs
**Media thickness to age**	Ctrl	-0.014	0.943	0.226	0.166	0.054
	TAV-ATAA	-0.192	0.328			
	BAV-ATAA	0.310	0.197			
**Media thickness to aortic diameter**	TAV-ATAA	-.048	0.81	nd	nd	0.266
	BAV-ATAA	0.151	0.537			
**Intima thickness to age**	Ctrl	-0.1	0.599	0.411	0.243	0.311
	TAV-ATAA	-0.038	0.848			
	BAV-ATAA	0.119	0.628			
**Intima thickness to aortic diameter**	TAV-ATAA	0.347	0.07	nd	nd	0.146
	BAV-ATAA	0.025	0.919			
	Ctrl	-0.057	0.763			

**Wall thickness to age**	TAV-ATAA<br>BAV-ATAA	-0.294<br>0.285	0.129<br>0.237	0.188	0.314	**0.031***
**Wall thickness to aortic diameter**	TAV-ATAA<br>BAV-ATAA	0.108<br>0.146	0.584<br>0.552	nd	nd	0.452
**Media thickness to intima thickness**	Ctrl<br>TAV-ATAA<br>BAV-ATAA	0.369<br>-0.394<br>0.088	0.045*<br>0.038*<br>0.721	**0.002****	0.172	0.057
**CD90+ cells in the media to age**	Ctrl<br>TAV-ATAA<br>BAV-ATAA	0.006<br>-0.204<br>0.066	0.973<br>0.298<br>0.789	0.222	0.424	0.197
**CD90+ cells in the media to aortic**	TAV-ATAA<br>BAV-ATAA	0.265<br>0.203	0.173<br>0.301	nd	nd	0.419
**Medial EF content to age**	Ctrl<br>TAV-ATAA<br>BAV-ATAA	0.115<br>0.287<br>-0.164	0.544<br>0.139<br>0.503	0.259	0.187	0.075
**Medial EF content to aortic diameter**	TAV-ATAA<br>BAV-ATAA	0.078<br>-0.127	0.691<br>0.605	nd	nd	0.260
**EF free area to age**	Ctrl<br>TAV-ATAA<br>BAV-ATAA	0.015<br>0.012<br>-0.061	0.939<br>0.953<br>0.805	0.496	0.405	0.410
**EF free are to aortic diameter**	TAV-ATAA<br>BAV-ATAA	0.474<br>0.625	0.011*<br>0.004**	nd	nd	0.248
**EF length to age**	Ctrl<br>TAV-ATAA<br>BAV-ATAA	-0.138<br>-0.186<br>0.078	0.467<br>0.343<br>0.75	0.430	0.246	0.203
**EF length to aortic diameter**	TAV-ATAA<br>BAV-ATAA	-0.249<br>0.024	0.201<br>0.922	nd	nd	0.192
**EF thickness to age**	Ctrl<br>TAV-ATAA	0.234<br>-0.299	0.213<br>0.122	**0.024***	0.301	0.116

	BAV-ATAA	0.074	0.764			
**EF thickness to aortic diameter**	TAV-ATAA<br>BAV-ATAA	-0.053<br>-0.149	0.788<br>0.542	nd	nd	0.381
**Lamellar units to age**	Ctrl<br>TAV-ATAA<br>BAV-ATAA	-0.139<br>0.133<br>0.047	0.465<br>0.499<br>0.85	0.162	0.277	0.393
**Lamellar units to aortic diameter**	TAV-ATAA<br>BAV-ATAA	-0.099<br>-0.276	0.615<br>0.253	nd	nd	0.283
**LUs/media thickness to age**	Ctrl<br>TAV-ATAA<br>BAV-ATAA	-0.112<br>0.376<br>-0.409	0.556<br>0.049*<br>0.082	**0.034***	0.154	**0.005****
**LUs/media thickness to aortic diameter**	TAV-ATAA<br>BAV-ATAA	0.08<br>-0.437	0.687<br>0.062	nd	nd	**0.043***
**Medial collagen content to age**	Ctrl<br>TAV-ATAA<br>BAV-ATAA	0.120<br>-0.051<br>-0.036	0.529<br>0.796<br>0.883	0.268	0.310	0.481
**Medial collagen content to aortic**	TAV-ATAA<br>BAV-ATAA	-0.016<br>-0.057	0.937<br>0.816	nd	nd	0.449
**Calcium content in the media to age**	Ctrl<br>TAV-ATAA<br>BAV-ATAA	-0.197<br>-0.530<br>0.605	0.297<br>0.004<br>0.006	**0.08***	**0.002****	**0.001****
**Calcium content in the media to aortic**	TAV-ATAA<br>BAV-ATAA	0.148<br>0.141	0.453<br>0.565	nd	nd	0.490
**OPN content in the media to age**	Ctrl<br>TAV-ATAA<br>BAV-ATAA	-0.076<br>0.161<br>0.069	0.693<br>0.433<br>0.785	0.204	0.328	0.389
**OPN content in the media to aortic**	TAV-ATAA<br>BAV-ATAA	0.396<br>0.422	0.045*<br>0.081	nd	nd	0.463

**Smooth muscle cells/0.01mm² in the media to age**	Ctrl	0.351	0.057	0.189	0.124	0.354
	TAV-ATAA	0.121	0.538			
	BAV-ATAA	0.002	0.994			
**Smooth muscle cells/0.01mm² in the**	TAV-ATAA	-0.04	0.84	nd	nd	0.213
	BAV-ATAA	0.212	0.384			
**Ki67+ Smooth muscle cells in the media/mm² to age**	Ctrl	0.035	0.856	0.325	0.159	0.084
	TAV-ATAA	-0.091	0.647			
	BAV-ATAA	0.337	0.158			
**Ki67+ Smooth muscle cells in the media/mm² to aortic diameter**	TAV-ATAA	0.222	0.256	nd	nd	**0.021***
	BAV-ATAA	-0.403	0.087			
**PDGF-BB in the media content to age**	Ctrl	0.416	0.025*	0.054	0.11	0.426
	TAV-ATAA	-0.007	0.972			
	BAV-ATAA	0.053	0.83			
**PDGF-BB content in the media to aortic**	TAV-ATAA	-0.157	0.425	nd	nd	0.396
	BAV-ATAA	-0.238	0.327			
**FGF-1 content in the media to age**	Ctrl	-0.15	0.429	0.213	0.246	0.493
	TAV-ATAA	0.072	0.722			
	BAV-ATAA	0.066	0.788			
**FGF-1 content in the media to aortic**	TAV-ATAA	-0.394	0.042*	nd	nd	**0.029***
	BAV-ATAA	0.192	0.431			
**TUNEL+/1000 cells in the media to age**	Ctrl	0.121	0.533	0.078	0.320	**0.044***
	TAV-ATAA	-0.277	0.171			
	BAV-ATAA	0.264	0.275			
**TUNEL+/1000 cells in the media to aortic**	TAV-ATAA	0.161	0.431	nd	nd	0.306
	BAV-ATAA	-0.003	0.992			

**α-SM-actin/Smooth muscel cell in the media to age**	Ctrl	-0.121	0.523	0.327	0.495	0.354
	TAV-ATAA	-0.241	0.217			
	BAV-ATAA	-0.125	0.61			
**α-SM-actin/Smooth muscle cell in the media to aortic**	TAV-ATAA	-0.23	0.238	nd	nd	0.151
	BAV-ATAA	-0.035	0.887			

2.3 Correlation analyses of intima thickness with media thickness as well gender specific analyses of media degeneration

The determination of the different layer thicknesses and their correlation analyses resulted in a negative correlation of intimal thickness with media thickness in TAV patients but no correlation in the control group or BAV patient group (Supplementary Figure S1a). In order to evaluate the influence of gender on the extent of media degeneration, groups were clustered in male and female and potential differences were statistically evaluated. Within the control group no gender dependent difference was observable. In contrast, the analysis if the extent of media degeneration of both aneurysm groups together revealed that female aneurysm patients were more affected by media degeneration than male patients (Supplementary Figure S1b). In addition, the severity of atherosclerotic changes is more pronounced in female aneurysm patients than male

patients, while no gender difference was detectable within the control group (Supplementary Figure S1c).

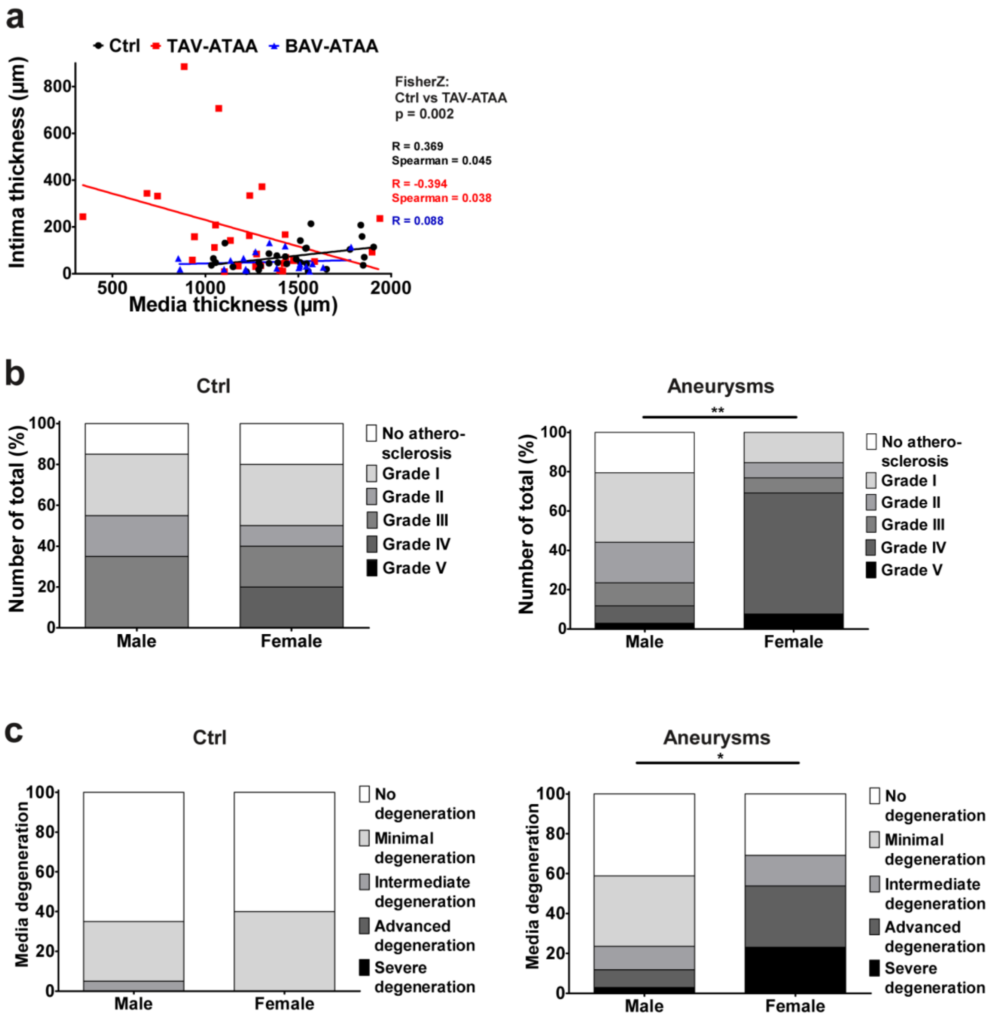

Figure S1: Correlation analyses of media and intima thickness as well as of gender specific differences in media degeneration. (a) Correlation of media with intima thickness was calculated using the spearman correlation coefficient and compared between Ctrl, TAV-ATAAs and BAV-ATAAs using Fisher-Z test. Results depicted in (b) shows the distribution of atherosclerotic changes and in (c) the distribution of media degeneration within the control and the aneurysm group in relation of the patient gender. (* <0.05, ** <0.01)

2.4 Determination of side specific differences in EF content and length as well as in the number of LUs

A statistical analysis of the side specific differences revealed a significantly higher EF content in the media near adventitia side compared to the media near intima side in the control group as well as in BAV- patients (Supplementary Figure S2a). In addition, the EF in both aneurysm patient groups are significantly longer in the media near adventitia side than in the media near intima side (Supplementary Figure S2b). The Supplementary Figure S2c shows that the absolute number of the LUs is reduced significantly in the TAV-ATAA group as compared to the control group. The calculation of the LU numbers in relation to the media thickness revealed no difference between the groups (Supplementary Figure S2d).

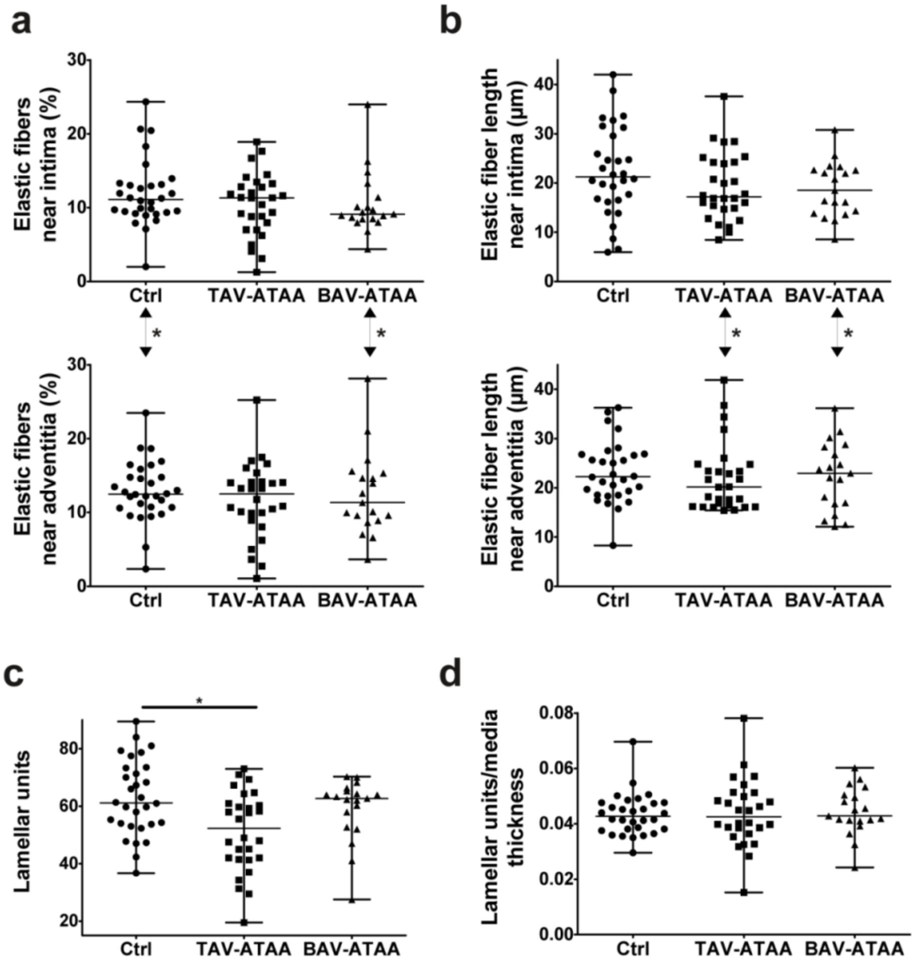

Figure S2: Detection of side specific differences in medial EF content and EF length as well as LU numbers. In (a) the side-specific evaluation of the amount of EF is shown and in (b) the same is shown for the EF length. In (c) the quantification of the absolute number of LUs is shown. In (d) the relative number of LUs in relation to the media thickness is depicted. (* <0.05)

2.5 Influence of pro-osteogenic factors on cellular calcium content and gender specific analyses of atherosclerosis grades

Incubation of primary smooth muscle cells with pro-osteogenic factors does not induce a calcium deposition *in vitro* (Supplementary Figure S3).

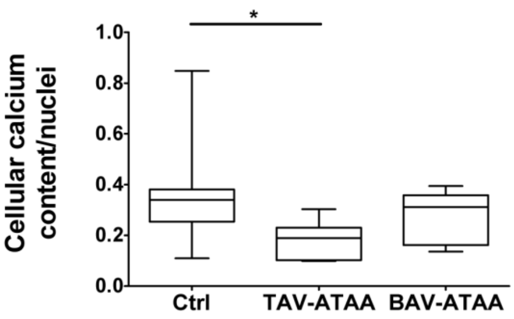

Figure S3: Determination of calcium depositions after incubation with pro-osteogenic factors in vitro as well as gender-dependent analysis of atherosclerosis severity. In (a) the quantification of calcium depositions within primary cell cultures after incubation with pro-osteogenic factors is shown. (* <0.05)

2.6 Analyses of side specific differences of Smooth muscle cell content and α-SM actin amount per cell

The comparison of the number of smooth muscle cells on the intima near and adventitia near media side with the Wilcoxon-rank-sum test revealed a reduced smooth muscle cell-density in the media near the intima side in control and in the in TAV group, but no such difference was observed in BAV-ATAA specimens (Supplementary Figure S4a). A reduction in the amount of α-SM actin per cell on both the intima and adventitia side of the media in the TAV group compared to the control group was also observed (Supplementary figure S4b). A significant side-specific difference in the α-SM actin expression per cell was only observed only in the BAV-ATAA group (reduced amount on the intimal side of the media; Supplementary Figure S4b).

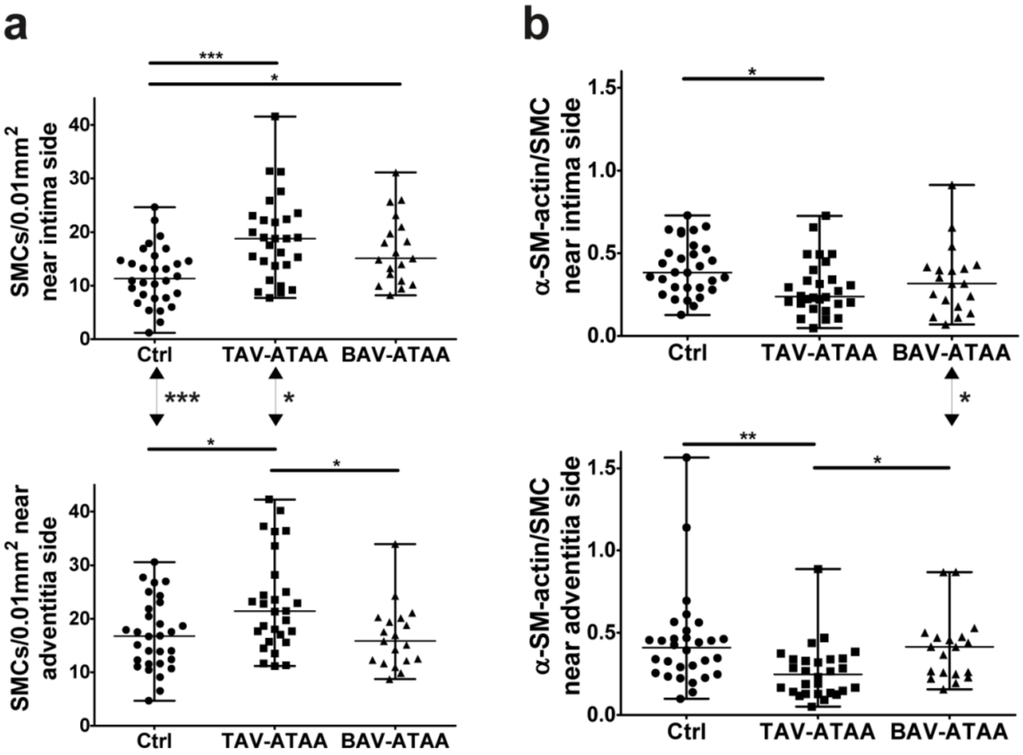

Figure S4: Detection of side specific differences in number of Smooth muscle cells and α-SM actin amount per cell. (a) shows the comparison of smooth muscle cell-number between the media near intima and the media near adventitia of the control and the two aneurysm groups. Likewise in (b) the side specific comparison of the α-SM actin amount per cell is depicted. (* <0.05, ** <0.01, ***<0.001)

2.7 Correction of statistical significant values for age and CHD

As the age and the proportion of patients with CHD differ significantly between the groups (please see Table 1), significant changes were corrected for these two factors. These evaluations were performed using ANCOVA analyses and the results are shown in Table S5. The columns "Group TAV vs. Ctrl", "Group BAV vs. Ctrl" and "Group TAV vs. BAV" gives the p-values of the corresponding contrast tests comparing the mean values of the

corresponding two-group comparisons. The column "Age" gives the p-values describing the correlation between age and the observed parameters over all three groups. The column "CHD" describes the results for testing the mean difference of observed parameters between patients with and without CHD. As depicted in Supplementary Table S5, influence of the age parameter on the result cannot be excluded for the number of smooth muscle cells per area.

Table S5: Statistical analysis of the influence of age and CHD on significant mean differences of the corresponding parameters. ANCOVA analyses including both co-variables age and CHD were performed (* <0.05, ** <0.01, *** <0.001)

	Ctrl vs	Ctrl vs	TAV- vs	Age	CHD
**Media thickness**	**0.002****	0.085	0.170	0.777	0.106
**Intima thickness**	**0.004****	0.553	**0.001****	0.412	0.874
**CD90+ cells in the**	**0.002****	0.521	**0.019***	0.486	0.702
**Calcium content in the**	0.235	0.769	0.161	0.645	0.594
**EF free area**	**0.001****	0.688	**0.007****	0.906	0.805
**Medial collagen content**	**0.005****	0.131	0.202	0.832	0.450
**OPN content in the media**	0.159	**<0.001*****	**0.033***	0.848	0.353
**Smooth muscle cells in the media/0.01mm2**	**0.015***	0.332	0.157	**0.029***	0.207
**Ki67+ smooth muscle cells in the media/mm2**	**0.016***	0.479	0.102	0.707	0.494
**α-SM-actin in the media/smooth muscle cell**	**0.023***	0.338	0.204	0.401	0.839
**Cleaved cellular/total OPN**	0.241	0.583	0.060	0.694	0.288

2.8 No evidence for a role of PDGF-BB and FGF-1 in increased smooth muscle cell proliferation and no evidence for medial smooth muscle death

Quantification of smooth muscle cell number revealed an increased density only in the aortic media of TAV-ATAA patients compared to the Ctrl group (Figure 5A). Of note, statistical evaluations gave that this parameter is also influenced by age of the patients (ODS Table S5). PDGF-BB and FGF-1 are well known pro-proliferative and migration-stimulating molecules for smooth muscle cells. However, as shown in Supplementary Figure S5a (quantification of staining for PDGF-BB, representative images in Supplementary Figure S5b) and Supplementary Figure S5c (quantification of staining for FGF-1, representative images in Supplementary Figure S5d) analyses did not provide evidence for differences in the expression or expression patterns of both molecules. Since an increase in smooth muscle cell proliferation, as observed in the TAV group, may indicate cell death and the need for repair, we tested for the occurrence of TUNEL positive smooth muscle cells, as well as cellular confluence over time, whereby no significant difference was observable (Supplementary Figure S5e,f).

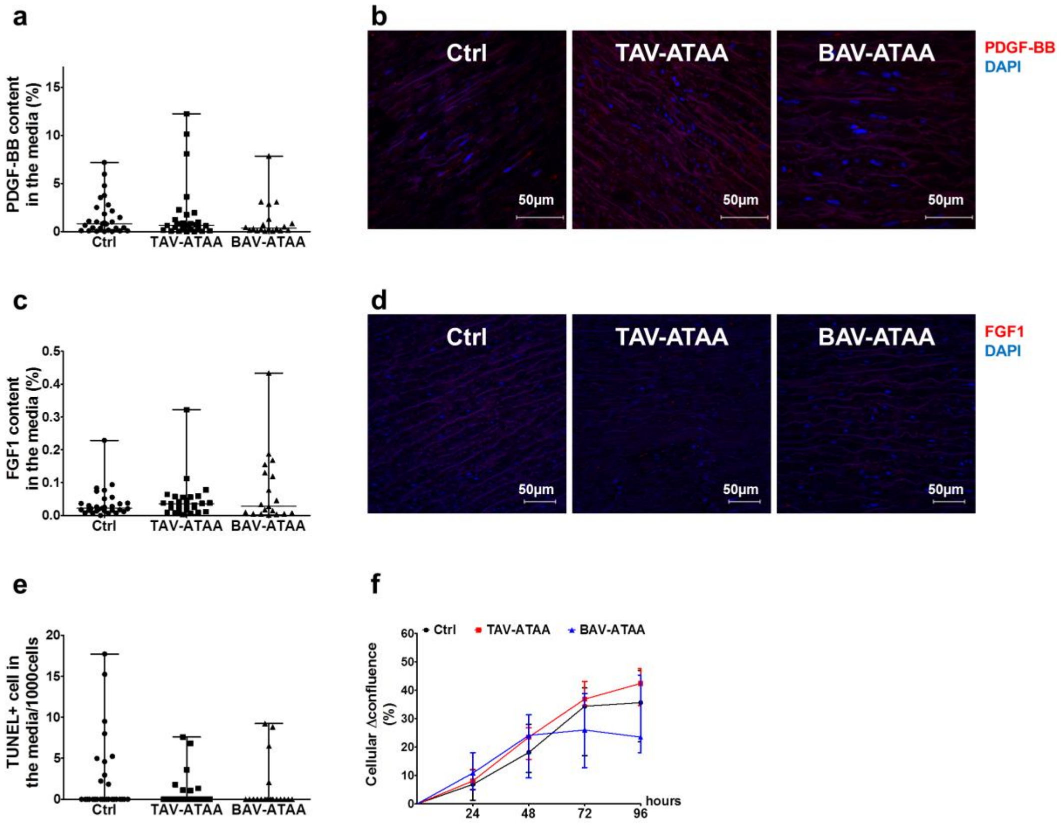

Figure S5: Determination of expression levels of the proteins PDGF-BB and FGF-1, determination of the number of Tunel positive cells as well as detection of cellular confluency of primary Smooth muscle cells in vitro over time. In (a) and (c) the quantification of PDGF-BB and FGF-1 expression within the aortic media is shown, respectively. Corresponding representative images are shown in (b) and (d) respectively (magnification 40x). Quantification of the amount of TUNEL positive smooth muscle cells within the aortic is shown in (e). (f) shows the determination of proliferation expressed as delta confluence of smooth muscle cells from Ctrl and aneurysmal tissue

REFERENCES

1 Prakash, S. K., Pedroza, C., Khalil, Y. A. & Milewicz, D. M. Diabetes and reduced risk for thoracic aortic aneurysms and dissections: a nationwide case-control study. *J Am Heart Assoc* **1**, doi:10.1161/JAHA.111.000323 (2012).

2 Losenno, K. L., Goodman, R. L. & Chu, M. W. Bicuspid aortic valve disease and ascending aortic aneurysms: gaps in knowledge. *Cardiol Res Pract* **2012**, 145202, doi:10.1155/2012/145202 (2012).
